# Factors Associated With Perception of Stigma Among Parents of Children With Cleft Lip and Palate: Cross-Sectional Study

**DOI:** 10.2196/53353

**Published:** 2024-03-04

**Authors:** Yanan Zhang, Xinwen Zhang, Jinzhuo Jiang, Wanhua Xie, Daoman Xiang

**Affiliations:** 1 Ophthalmology and otorhinolaryngology Department Guangzhou Women and Children's Medical Center, Guangzhou Medical University Guangdong Provincial Clinical Research Center for Child Health Guangzhou China; 2 College of Engineering South China Agricultural University Guangzhou China; 3 Outpatient Department Guangzhou Women and Children's Medical Center, Guangzhou Medical University Guangdong Provincial Clinical Research Center for Child Health Guangzhou China

**Keywords:** stigma, social anxiety, depression, parents of children with cleft lip and palate, cleft lip, cleft palate, cross-sectional study

## Abstract

**Background:**

Parents of children with cleft lip with or without cleft palate (CL/P) often face stigmatization, which has a significant impact on their quality of life and mental health. However, to date, there is a lack of comprehensive, multicenter empirical research on parents of children with CL/P in China, particularly those with large-scale samples.

**Objective:**

This study aimed to identify major factors that contribute to the perception of stigma experienced by parents of children with CL/P.

**Methods:**

A cross-sectional survey was conducted. A total of 104 parents of children diagnosed with CL/P in 2 hospitals were selected by convenience sampling. Demographics and disease information, the Chinese Perception of Stigma Questionnaire, the Center for Epidemiological Studies Depression Scale, and the Social Anxiety Scale were used in this study. Descriptive statistics, *t* tests, and one-way ANOVA were used to compare the differences between participants’ demographic information and perception of stigma. Multivariable linear regression was performed to assess associations between demographic factors, social anxiety, depression, and perception of stigma.

**Results:**

The mean scores for the dimensions of perception of stigma, depression, and social anxiety were 22.97 (SD 9.21), 38.34 (SD 8.25), and 22.86 (SD 6.69), respectively. Depression and social anxiety were positively associated with discrimination, while surgery status was a negatively associated variable. Parents with a college education or higher had significantly lower levels of perceived stigma compared to parents with a junior high school education (all *P* values <.05). These 4 factors explained 40.4% of the total model variance (*F*_8_=9.726; *P*<.001; *R*^2^=0.450; adjusted *R*^2^=0.404).

**Conclusions:**

Our findings highlight a concerning trend of diminished quality of life among parents of children with CL/P. Factors such as parents’ education level, surgery status, depression, and social anxiety are shown to influence the level of stigma experienced. Implementing comprehensive nursing care and providing presurgical support are effective strategies for alleviating parents’ social anxiety, reducing perceived stigma, and preventing depression.

## Introduction

Cleft lip with or without palate (CL/P) is a common congenital developmental malformation affecting the oral and maxillofacial region [1], with a global prevalence of about 1.62%-1.82% [2], and the highest prevalence in Asian countries [3]. There are 35,000 babies born with CL/P in China each year [4]. Palate repair surgery is usually done when babies are 10 to 24 months old, while lip surgery is generally performed at the age of 6 months [3]. The management of CL/P is long-term, beginning from birth and continuing into the late teenage years. Children born with CL/P not only face a visible facial disfigurement but also have other challenges related to the cleft, such as feeding difficulties, speech impediments, hearing issues, and psychological problems, which may compromise their ability to communicate effectively [5].

Children with CL/P are vulnerable to external discrimination because of the malformation. A child with CL/P is subjected to bullying, rejection, and social isolation, sometimes even from their own family [6]. The discrimination can have devastating health and well-being impacts, not only on children affected but also on people associated with the discriminated person. Having a baby with CL/P has variable psychological impacts on the parents [7]. Parents are often concerned about whether their children will be accepted as they grow up, and parents’ psychological state directly affects their children’s perception of the condition [6,8,9]. Some studies [10,11] have indicated that the perception of stigma experienced by the mother and children is consistent. Numerous studies [2,12,13] have shown that children with CL/P experience significant stigma, and limited engagement in social activities suggests that the parents of these children may experience high levels of stigma. Furthermore, Parents are troubled by their children’s conditions from birth to adulthood, while children often experience discrimination after their cognitive development has matured. Therefore, exploring ways to prevent these patients and their parents from feeling stigmatized is urgently necessary.

Currently, research on the perception of stigma among parents of children with CL/P is predominantly qualitative, emphasizing the exploration of factors contributing to parental stigma. However, there is limited reporting on assessments regarding the level and variation in the perception of stigma. In particular, there is no research on the perception of stigma in children with CL/P in China. Previous studies [11,14] have shown that superstition is the main reason for parents’ perception of stigma, and only 40% of parents believe that genetic problems cause CL/P. One study [11] found that 72% of mothers felt ashamed of having children with CL/P, and some even abandoned their babies. Therefore, it is crucial to examine the perception of stigma among parents of children with CL/P, pinpoint those experiencing heightened stigma levels, implement tailored nursing interventions based on sensitive factors, and mitigate the likelihood of public incidents. This study aims to investigate experiences of stigma in parents of children with CL/P and analyze the contributing factors.

## Methods

### Study Design and Participants

This cross-sectional study was conducted using a convenience sample of parents of children with CL/P, recruited from 2 hospitals in southern and western China (from the cities of Guizhou and Guangzhou) between June 2020 and January 2023. All parents of children who met the inclusion and exclusion criteria for surgical treatment in the Department of Stomatology were invited to complete a questionnaire.

The inclusion criteria were as follows: parents of children (aged <15 years) with CL/P undergoing nasoalveolar molding therapy at the Department of Stomatology. These parents were invited to complete an anonymous questionnaire.

Exclusion criteria included parents of infants with additional birth defects or medical conditions, those with genetic diagnoses (eg, Down syndrome or Trisomy 21 syndrome), and individuals unable to complete the questionnaires due to critical illness or mental disorders.

### Data Collection

After the doctors introduced the operational procedure or the nurses introduced the environment of the wards, the participants were invited to fill in the questionnaires. Uniform instructions were used to explain the study aims and their relevance to the participants. The participants were informed that the study was anonymous and voluntary and that they could quit anytime.

### Variables and Instruments

The demographic data were collected via a questionnaire. Three questionnaires with good reliability and validity were used to collect the data on stigma, social anxiety, and depression.

#### Demographics Questionnaire

The demographic information questionnaire was designed by researchers after consulting the literature and mainly included parents’ age, role (father or mother), education level, and working status as well as children’s age, sex, type of CL/P, surgery status, and number of surgeries.

#### Disability Discrimination Perception Scale

Stigma was assessed using the Disability Discrimination Scale (DDPS), initially developed by Liu and Shen [15]. The questionnaire comprises 10 items (eg, “When participating in activities, I feel that people around me would not talk to me and avoid me”), designed to explore the perception of stigma among individuals with disabilities in their daily lives. It is also suitable for children aged 10 to 16 years. Respondents rated each item on a 5-point scale, ranging from 1 (very inconsistent) to 5 (completely consistent). Higher total scores indicate a greater perception of stigma, with scores ranging from 10 to 50. The scale demonstrates good internal consistency, as evidenced by a Cronbach α reliability of 0.89. The criterion validity was 0.47, indicating good validity [15].

#### The Center for Epidemiological Studies Depression Scale

The level of depression was measured by the Center for Epidemiological Studies Depression Scale (CES-D) [16]. CES-D consists of 20 items (eg, “I was bothered by things that usually don’t bother me”), and each item is scored from 0, indicating “rarely or none of the time (less than 1 day)” to 3, indicating “most or all of the time (5-7 days).” The total score of all items ranges from 12 to 60, with higher scores indicating a greater likelihood of depression; a total score of 15 points or less indicates no depression; 16 to 19 points indicate possible depression; and a score of 20 points or higher indicates definite depression [16]. The scale is suitable for screening people with depressive symptoms and can also be used to assess the severity of depressive symptoms. The Cronbach α of CES-D was 0.87, indicating good internal consistency. The split-half reliability was 0.85, the test-retest reliability was 0.70, and the criterion validity was 0.75. Therefore, CES-D had good reliability and validity.

#### The Interaction Anxiousness Scale

The Interaction Anxiousness Scale (IAS) was used to assess the subjective propensity to experience social anxiety independent of behavior [17]. The IAS contains 15 self-reported items (eg, “I want to be confident in social situations more”), which are answered on a 5-point scale, ranging from 1 (very inconsistent) to 5 (completely consistent). Total scores range from 15 to 75, with higher scores indicating higher levels of social concern. The Cronbach α reliability of the scale was 0.87, the test-retest reliability was 0.80, and the criterion validity was 0.48. In this study, according to the actual situation, 7 items related to sexual life were deleted, and 8 items were selected for evaluation. The reliability of the scale was retested, and Cronbach α reliability was 0.68, indicating acceptable reliability.

### Statistical Analysis

IBM SPSS Statistics (version 25.0; IBM Corp) for Windows, was used for statistical analysis. Standard descriptive statistical values (means and SDs) were calculated. The independent samples 2-tailed *t* test and ANOVA were used for the intergroup comparisons of parameter changes. Pearson analysis was used to examine the relationship between perception of stigma, social anxiety, and depression. Multiple linear regression was performed to assess the association between demographic factors, anxiety, depression, and perception of stigma. The significance level was set at 5%.

**Ethical Considerations**

The study was approved by the Ethics Committee for Clinical Studies at Guangzhou Women and Children's Medical Center, Guangzhou Medical University (NO.2022268A01). This study obtained informed consent from participants, who had the option to withdraw at any time. The research data were collected anonymously.

## Results

In this study, a total of 110 questionnaires were distributed. Of the collected questionnaires, 104 were complete, resulting in a 94.6% response rate.

### Participant Characteristics

Most participants were mothers aged 25-30 years and unemployed, with a high school diploma. Their children had CL/P, and surgery had not been performed. Unemployed parents constituted 71% (n=74) of the sample, with nearly half (n=14, 47%) of the employed parents being self-employed. Over half (n=61, 59%) of the respondents had completed high school. The children’s average age was 3.06 (SD 2.02) years. A total of 73 (72%) children had not undergone surgery, while most of those who had done the surgery (n=20, 65%,) had undergone the procedure twice. A descriptive analysis of the general demographics, work-related characteristics, and disease-related information of the participants is shown in Table 1.

**Table 1:** Demographic characteristics of the participants (N=104).

Variable and category		n (%)
**Age (years)**		
	18-25	30 (29)
	25-30	42 (40)
	30-35	26 (25)
	＞35	6 (6)
**Role**		
	Father	39 (38)
	Mother	65 (63)
**Educational background**		
	Primary school or lower	6 (6)
	Junior high school	23 (22)
	Senior high school	61 (59)
	College	7 (7)
	Bachelor’s degree or higher	7 (7)
**Working status**		
	Unemployed	74 (71)
	Working	30 (29)
**Occupation**		
	Unemployed	74 (71)
	Independent management	14 (14)
	Enterprise unit	9 (9)
	Public service unit	7 (7)
**Children’s sex**		
	Male	60 (58)
	Female	44 (43)
**Condition type**		
	Cleft lip	36 (35)
	Cleft lip and palate	68 (65)
**Number of surgeries**		
	0	73 (70)
	1	7 (7)
	2	20 (19)
	≥3	4 (4)
**Status of surgery**		
	After surgery	73 (70)
	Before surgery	31 (30)

### Prevalence of Stigma, Social Anxiety, and Depression

The mean scores for the dimensions of perception of stigma, depression, and social anxiety were 22.97 (SD 9.21), 38.34 (SD 8.25), and 22.86 (SD 6.69), respectively. Additionally, 38% (n=40) of the participants exhibited a perception of stigma level beyond the moderate level, and every participant experienced evident depression, with 72% (n=75) displaying average levels of social anxiety.

### Univariate Analyses of Factors Associated With Stigma

The demographic data, including parents’ age, role, education level, and working status as well as the children’s age, sex, CL/P type, surgery status, and the number of surgeries, were analyzed using a one-way ANOVA and *t* tests. The results of the Kolmogorov-Smirov test suggested that the continuous variables were reasonably and normally distributed. The *t* tests revealed that the parents whose children had undergone surgery experienced lower stigma levels compared to those whose children had not undergone surgery (*P*=.01). The perception of stigma scores differed significantly among parents having different educational backgrounds (*P*<.001) and between those whose children underwent different numbers of surgeries (*P*=.04). The specific results are detailed in Table 2.

**Table 2:** Univariate analyses of factors associated with stigma (N=104).

Variable and category		Mean (SD)	*F* test (*df*)	*t* test (*df*)	*P* value
**Age (years)**			1.58 (3)	—^a^	.20
	18-25	25.74 (9.93)			
	25-30	21.14 (8.46)			
	30-35	22.24 (8.30)			
	>35	23.50 (12.06)			
**Role**			—	0.32 (102)	.75
	Father	23.28 (9.62)			
	Mother	22.69 (8.96)			
**Education background**			3.62 (4)	—	<.001
	Primary school or lower	22.50 (9.89)			
	Junior high school	24.52 (8.94)			
	Senior high school	24.21 (9.34)			
	College	16.14 (2.73)			
	Bachelor’s degree or higher	13.43 (1.81)			
**Working status**			—	2.02 (102)	.06
	Unemployed	24.00 (8.84)			
	Working	20.23 (9.56)			
**Occupation**			1.36 (3)	—	.26
	Unemployed	24.00 (8.84)			
	Independent management	19.43 (8.49)			
	Enterprise unit	21.89 (10.95)			
	Public service unit	19.71 (10.95)			
**Children’s sex**			_—_	0.83 (102)	.41
	Male	23.55 (9.47)			
	Female	22.05 (8.78)			
**Condition type**			—	0.34 (102)	.74
	Cleft lip	23.33 (8.91)			
	Cleft lip and palate	22.69 (9.36)			
**Number of surgeries**			2.94 (3)	—	.04
	0	24.34 (9.49)			
	1	17.57 (7.28)			
	2	18.95 (7.28)			
	≥3	26.00 (7.07)			
**Status of surgery**			—	2.50 (102)	.01
	Before surgery	24.34 (9.49)			
	After surgery	19.55 (7.47)			

^a^Not applicable.

### Correlation Analysis With Perception of Stigma, Social Anxiety, and Depression

The results showed that the perception of stigma was related to social anxiety (*r*=0.54; *P*<.001) and depression (*r*=0.39; *P*<.001); there was also a correlation between depression and social anxiety (*r*=0.30; *P*=.002). The specific results are shown in Table 3.

**Table 3:** Correlation analysis (Pearson r) with stigma, social anxiety, and depression. At *P*=.01 (2-tailed), the correlation was significant.

Variable		Stigma	Depression	Social anxiety
**Stigma**				
	*r*	1	0.39	0.54
	*P* value	—^a^	<.001	<.001
**Depression**				
	*r*	0.39	1	0.30
	*P* value	<.001	—	.002
**Social anxiety**				
	*r*	0.54	0.30	1
	*P* value	<.001	.002	—

^a^Not applicable.

### Regression Analyses of Stigma

With the perception of stigma as the dependent variable, depression, social anxiety, and demographic data with differences between groups were included in the model as independent variables, and multiple linear regression analyses were performed. In the perception of stigma model, depression, social anxiety, educational background, and status of surgery were significant correlates explaining 40.4% of the total model variance (*F*_8_=9.726; *P*<.001; *R*^2^=0.450; adjusted *R*^2^=0.404). Depression and anxiety were positively associated with discrimination, while the status of surgery was a negatively associated variable. Parents of children with a college education or higher had significantly lower levels of perceived stigma compared to parents with a junior high school education (all *P* values <.05). There were no significant differences in the perceived stigma levels between parents with elementary or high school education and those with junior high school education (all *P* values >.05). The specific results are shown in Table 4.

**Table 4:** Regression analyses of stigma.

Variables	Unstandardized coefficients (B)	SE	*t* test (*df*)	*P* value
Constant value	0.10	4.36	0.02 (95)	.98
Status of surgery	–5.85	2.22	–2.64 (95)	.01
Number of operations	2.08	1.14	1.82 (95)	.07
Depression	0.26	0.09	2.93 (95)	<.001
Social anxiety	0.52	0.12	4.48 (95)	<.001
Primary school or lower	0.23	3.30	0.07 (95)	.95
Senior high school	–0.29	1.75	–0.16 (95)	.87
College education	–6.53	3.09	–2.11 (95)	.04
Bachelor’s degree or higher	–7.21	3.15	–2.29 (95)	.02

## Discussion

This study found that parents of children with CL/P experienced a high level of perception of stigma and obvious social anxiety and depression. In addition, parents’ education level, children’s status of surgery, social anxiety, and depression affect parents’ levels of perceived stigma. This study complements the gap in the study of stigma perception in families of children with CL/P in China. Parents in our study exhibited a higher level of stigma perception. This could be attributed to the fact that most children underwent corrective surgery during infancy, making parents more attuned to discrimination, as they believe that visual impairments affect their children’s social lives. Thus, prevailing data suggest that Chinese clinical nurses should pay attention to the quality of life of parents of children with CL/P.

Our study showed that children’s sex and the type of CL/P could not affect parents’ discrimination perception. This is consistent with the findings of previous studies that reported no significant difference in the level of discrimination perception between patients of different sexes and CL/P types [10]. The results of our study showed no significant difference in the level of stigma perception between fathers and mothers. Fathers also had a high level of stigma perception. However, previous studies [14,18] suggested that mothers of children with CL/P experienced severe stigma or discrimination while ignoring the possibility that fathers also experienced high levels of discrimination. Two other studies [19,20] pointed out that the main sources of stress among fathers were the treatment process, feeding, and social stigma. Therefore, in the care process of children with CL/P, we should not only focus on the mother’s psychological state but also consider the psychological state of the father. The importance of family nursing should also be emphasized.

Parents of children with CL/P experience obvious depression and anxiety. It is consistent with the findings of previous studies reporting that parents of newborns with CL/P had significantly higher levels of anxiety compared to control parents [20]. The study findings are consistent with the current literature, as depression or depressed mood in parents of children with CL/P is a common phenomenon [21,22]. Kumar [23] reported the highest incidence of depressive episodes, with 42% of parents of children with CL/P aged >10 years showing a strongly or very strongly elevated depressive disorders screening index. Sommer [24] has pointed out that mothers of children with CL/P believe their children have a negative impact on them. Their negative views and emotions are mainly derived from the long treatment times, the difficulty of treatment, the uncertainty of the infant’s future condition, and the fear of rejection. Family members of infants with CL/P experience stigma, anxiety, and worry, which may lead to individual negative emotions and coping styles, produce adverse intergroup relations, reduce subjective well-being and life satisfaction, and ultimately affect the quality of family life. Relieving the stigma perceived by parents of children with CL/P is the first step to relieving negative emotions like anxiety and depression in these parents. Therefore, we should pay attention to the physical rehabilitation of children as well as the mental health of children and their parents.

In this study, parents with junior high school education showed a high level of stigma perception, and the perception of stigma was lower among parents with a college education or higher. So far, no study has investigated the relationship between the education level of parents who have children with CL/P and their stigma perception. A previous study [6] showed that caregivers of children with attention deficit hyperactivity disorder, who have higher education levels, experience higher levels of stigma. However, the results of this study were inconsistent with a previous study, possibly because parents with a junior high school education have less knowledge of the condition and are more likely to focus on the appearance of their children. The difference could potentially be attributed to the fact that parents with higher education levels are more likely to access relevant information about the condition and feel more confident about the later recovery process. Hence, health care professionals should focus on parents with a high school education or lower, monitor variations in their perceived stigma levels, comprehend their psychological well-being across different stages of their children’s lives, and offer tailored psychological support.

Cleft surgery provides hope to those children and their parents, offering the possibility of re-enrollment in schools, employment opportunities, social acceptance, and improved prospects for marriage [6]. Undergoing surgery directly affects the level of the stigma perceived by parents. Parents of children without surgery perceive a higher level of stigma, which is consistent with previous studies [14] suggesting that parents of children with CL/P show obvious negative emotions in the first 3 months after the birth of their children without surgery. Therefore, the initial 3 months emerge as a pivotal phase for health care professionals to alleviate parental anxiety and deliver emotional and educational assistance to parents of infants with CL/P. Through comprehensive nursing and presurgical support, mothers can enhance their early feeding capabilities, expedite the bonding process with their infants, and facilitate the infants’ swift recovery. Familiarity with CL/P beforehand serves to diminish maternal stress during initial interactions, mitigating anxiety and perceptions of discrimination. Consequently, encouraging mothers to engage with CL/P-related charitable organizations or fostering interaction among children with CL/P in shared spaces can effectively reduce anxiety and perception of stigma levels.

This study has several limitations. All the participants in this study were family members of children who were willing to undergo surgery, and no family members of children who were unwilling to undergo treatment were included, which may have led to selective bias. Parents filled out the scale based on recall, which may introduce information bias. The sample size of this study was small, as it was limited to hospitals only from 2 provinces with a large gap in economic levels between the 2 places, and fewer fathers were included in the study. Future studies should increase the sample size and broaden the scope of the investigation.

Our findings highlight a concerning trend of diminished quality of life among parents of children with CL/P. Factors such as parents’ education level, the status of surgery in children, depression, and social anxiety are shown to influence the level of stigma experienced by these parents. Implementing comprehensive nursing care and providing presurgical support are effective strategies for alleviating parents’ social anxiety, reducing perceived stigma, and preventing depression.

## Abbreviations

CES-D: Center for Epidemiological Studies Depression Scale
CL/P: cleft lip with or without cleft palate
DDPS: Disability Discrimination Scale
IAS: Interaction Anxiousness Scale

## Data Availability

The data sets generated and analyzed during this study are available from the corresponding author upon reasonable request.
